# Isolation of a dengue type 1 virus from a soldier in West Africa (Côte d'Ivoire)

**DOI:** 10.3201/eid0601.000116

**Published:** 2000

**Authors:** J P Durand, L Vallée, J J de Pina, H Tolou


### Isolation of a Dengue Type 1 Virus from a Soldier in West Africa (Côte d'Ivoire)

**To the Editor:** In Africa, recent reports of epidemic or endemic dengue activity usually come from the eastern part of the continent (1), and the serotype most frequently identified is dengue 2.

We report the isolation of a dengue 1 virus strain from the blood of a young soldier living in Abidjan, the capital of Côte d'Ivoire. This 20-year-old man arrived from France on October 19, 1998. On December 28, 1998, he consulted a physician of his regiment because of headache, diarrhea, and fever (40°C). The results of the laboratory investigations were as follows: platelets (193 $10^9$ /L), leukocytes (2.210$^9$ /L); no malaria was found. He was hospitalized for possible arboviral infection, and treatment with paracetamol was prescribed. On December 29, a blood sample was collected; serum and buffy coat were frozen at -20°C for further examination at the Institute of Tropical Medicine of the Military Health Services. On day 3, the patient's temperature dropped to 38°C, then rose to 39.5°C on day 5. All symptoms were resolved on day 6.

On February 15, 1999, the frozen blood was defrosted and the lysed buffy coat was immediately cocultured with C6/36 cells. On day 6, a blind passage was made on the same cells and on Vero. On day 12, no cytopathic effect was observed, but a dengue 1 virus was identified by indirect fluorescent antibody assay (2) with type-specific monoclonal antibodies. This diagnosis was confirmed by reverse transcription-polymerase chain reaction, with a technique slightly modified from Lanciotti (3).

On the first blood specimen, the serologic immunoglobulin (Ig)M assays (M antibody capture enzyme-linked immunosorbent assay [ELISA]) with our antigen screening panel were negative (dengue, West Nile, Chikungunya, Rift Valley fever). The IgG assay (ELISA) against dengue antigen was also negative.

The patient returned to France in February 1999. A second blood sample was collected and tested on April 7, 1999, 3 months after the illness. The serologic assays were positive against the dengue antigens at the following dilutions of the serum: IgM: dengue 1: 1/120,000; dengue 2: 1/40,000; dengue 3: 1/12,000; dengue 4: 1/25,000. IgG: dengue 1: 1/75,000; dengue 2: 1/90,000; dengue 3: 1/60,000; dengue 4: 1/120,000. This seroconversion allowed us to confirm infection of this patient by a dengue virus.

The lack of similar reported cases or epidemics among the local and expatriate populations of Abidjan may indicate poor transmission, recent introduction of the strain, or low virulence of the virus, as previously hypothesized for dengue in West Africa (4). However, the serologic status of the human population needs to be further investigated. Characterization of the isolated viral strain would be of interest for dengue epidemiology. Complete sequencing of the viral RNA is in progress in our laboratory.

Human infection with dengue virus has been rarely reported and studied in West Africa, and the epidemiology of serotype 1 is poorly documented. During the past 35 years, the Pasteur Institute of Dakar confirmed three dengue 1 strains (two from humans in Senegal; one from mosquitoes in Côte d'Ivoire), while during the same period, more than 300 dengue 2 strains were studied, most from mosquitoes (5). In the past 10 years, medical and entomologic surveys showed circulation of dengue 2 virus in Senegal (one isolate in the blood of a French soldier) (6). However, during the 1970s, Nigerian virologists demonstrated circulation of dengue 1 and 2 in their country: more than 50% of the adults living in the savannah had neutralizing antibodies (7). Of 148 blood samples of febrile patients, three viral strains (yellow fever, dengue 1, and Zika) were isolated, all from children (8).

In Africa, outside the epidemics of yellow fever, it is difficult to isolate arboviruses from adult humans. Isolation is often more successful from children or naive expatriates. Accordingly, soldiers participating in international operations constitute a very exposed population. During recent operations in Somalia (1992-93), dengue fever was an important cause of febrile illness among U.S. troops (9). Thirty-nine dengue 2 and three dengue 3 strains were isolated from 96 collected sera.

### Acknowledgments

We thank F. Tock and S. Guesdon for technical assistance and D. Gubler and N. Karabatsos for the monoclonal antibodies used for virus typing.

This work was supported by the Délégation Générale à L'Armement.

**J.P. Durand,* L. Vallée,† J.J. de Pina,‡ and H. Tolou***
Institut de Médecine Tropical du Service de Santé des Armées, Marseille Armées, France; †Service Médical du 43 BIMa, Abidjan, République de Côte d'Ivoire; and ‡Hôpital Laveran, Marseille Armées, France

### References

1. Gubler DJ, Kuno G. Dengue and dengue hemorrhagic fever. Center for Agriculture and Biosciences International; 1997; p. 10-25.
2. Henchal EA, Gentry M, McCown JM, Brandt WE. Dengue virus specific and flavivirus group determinants identified with monoclonal antibodies by indirect immunofluorescence. Am J Trop Med Hyg 1982;31:830-6.
3. Lanciotti RS, Calisher CH, Gubler DJ, Chang GJ, Vorndam AV. Rapid detection and typing of dengue viruses from clinical samples by using reverse transcriptase-polymerase chain reaction. J Clin Microb 1992;30:545-51.
4. Deubel V, Nogueira RM, Drouet MT. Direct sequencing of genomic cDNA fragments amplified by the polymerase chain reaction for molecular epidemiology of dengue 2 virus. Arch Virol 1993;129:197-210.
5. Centre Collaborateur OMS de Reference et de Recherche pour les Arbovirus et les Fièvres Hémorragiques. Rapport Annuel 1995; Institut Pasteur de Dakar.
6. Monlun E, Zeller H, Le Guenno B, Traore-Lamizana M, Hervy JP, Ferrara L, et al. Surveillance of the circulation of arbovirus of medical interest in the region of eastern Senegal. Bull Soc Pathol Exot 1993;86:21-8.
7. Fagmani A. Epidemiological investigations on arbovirus infections at Igbo-Ora, Nigeria. Tropical and Geographical Medicine 1977;29:187-91.
8. Fagmani AH, Monath TP, Fabiyi A. Dengue virus infection in Nigeria: a survey for antibodies in monkeys and humans. Trans R Soc Trop Med Hyg 1977;71:60-5.
9. Sharp TW, Wallace MR, Hayes CG, Sanchez JL, DeFraites RF, Arthur RR, et al. Dengue fever in U.S. troops during Operation Restore Hope, Somalia, 1992-1993. Am J Trop Med Hyg 1995;53:89-94.

### Carbapenem-Hydrolyzing Metallo-ß-Lactamase from a Nosocomial Isolate of *Pseudomonas aeruginosa* in France

**To the Editor:** The carbapenems (meropenem and imipenem), the ß-lactams with the broadest spectrum, are stable to most ß-lactamases (1). Therefore, they are often used as antibiotics of last resort for treating nosocomial infections due to gram-negative bacteria resistant to other ß-lactams. Resistance to carbapenems and susceptibility to other ß-lactams in *Pseudomonas aeruginosa* is common as a result of reduced drug accumulation or increased expression of pump efflux (1).

Several extended-spectrum ß-lactamases have been reported in *P. aeruginosa*, but only two, IMP-1 and VIM-1, possess an extended hydrolysis profile that includes carbapenems (2-5). The chromosome-borne and plasmid-mediated carbapenem-hydrolyzing ß-lactamase, IMP-1, has been described in several gram-negative rods, including *P. aeruginosa*, *P. cepacia, Alcaligenes xylosoxydans,* and *Enterobacteriaceae* isolates in Japan (4,6). Recently, a chromosome-borne carbapenem-hydrolyzing ß-lactamase, VIM-1, was reported from a clinical isolate of *P. aeruginosa* in Italy (5), and uncharacterized carbapenem-hydrolyzing ß-lactamases have been reported in the United Kingdom and Portugal (7,8). The weakly related IMP-1 and VIM-1 (31.4% amino acid identity) are both zinc-dependent (metallo-enzymes) and confer resistance to all ß-lactams except monobactams (3,5).

In 1996, a 39-year-old French woman was hospitalized in Marseille for chronic myelogenous leukemia, pancytopenia, and allogeneic bone marrow transplantation. After a 15-day stay in the transplantation unit, fever developed and imipenem and amikacin were administered. Despite this treatment, the patient died of septic shock syndrome 5 days later. Three-day-old blood cultures grew a carbapenem-resistant *P. aeruginosa* isolate. This *P. aeruginosa* COL-1 isolate was resistant to most ß-lactams, including piperacillin/tazobactam, imipenem, meropenem, ceftazidime, cefepime (minimum inhibitory concentrations [MICs] of 128, 32, 16, 64, 32 mg/L, respectively), amikacin, tobramycin, gentamicin, netilmicin, and ciprofloxacin; however, the isolate was susceptible to aztreonam (MIC determination, genetic techniques and ß-lactamase assays are described elsewhere [9]). A sonicate of crude extract of *P. aeruginosa* COL-1 culture showed strong imipenem and meropenem hydrolysis activity (0.7 mU/mg and 1.9 mU/mg; reference *P. aeruginosa* strain <0.05 mU/mg) by UV spectrophotometry with 0.1 mM of substrate, after incubation in 50 mM phosphate buffer at 30°C. This activity was lost when the enzyme extract was preincubated with 10 mM of edetic acid and was partially restored

